# Statin use and fall risk in adults: a cross-sectional survey and mendelian randomization analysis

**DOI:** 10.3389/fphar.2024.1364733

**Published:** 2024-06-26

**Authors:** Hui Zheng, Yong-Jiang Fang, Shu-Ting Wang, Yan-Bing Huang, Tai-Chun Tang, Min Chen

**Affiliations:** ^1^ Acupuncture and Tuina School, Chengdu University of Traditional Chinese Medicine, Chengdu, China; ^2^ Department of Acupuncture, Kunming Municipal Hospital of Traditional Chinese Medicine, Kunming City, China; ^3^ Department of Colorectal Diseases, Hospital of Chengdu University of Traditional Chinese Medicine, Chengdu, China

**Keywords:** fall risk, balance problem, statin use, cross-sectional study, mendelian randomization analysis

## Abstract

**Background and Objective:**

The issue of falls poses a significant threat to the health of the elderly population. Although statins can cause myopathy, which implies that they may cause balance problems and increase the risk of falling, this has not been tested. Our objective was to assess whether the use of statins is linked to a higher risk of falls.

**Methods:**

A cross-sectional survey study and Mendelian randomization (MR) study were conducted to examine whether the use of statins was associated with an increased risk of falling and balance problems. The cross-sectional study included 2,656 participants from the US population (NHANES) who reported information on balance and falling problems in the past year and their use of statins. Univariate and multivariate logistic regression models were used to investigate the association between statin use and the likelihood of falling or experiencing balance problems. The MR study identified five Single Nucleotide Polymorphisms (SNPs) that predict statin use across five ancestry groups: Admixed African or African, East Asian, European, Hispanic, and South Asian. Additionally, SNPs predicting the risk of falls were acquired from the UK Biobank population. A two-sample MR analysis was performed to examine whether genetically predicted statin use increased the risk of falls.

**Results:**

The use of statins was found to be associated with an increased likelihood of balance and falling problems (balance problem, OR 1.25, 95%CI 1.02 to 1.55; falling problem, OR 1.27, 95%CI 1.03–1.27). Subgroup analysis revealed that patients under the age of 65 were more susceptible to these issues when taking statins (balance problem, OR 3.42, 95%CI 1.40 to 9.30; falling problem, OR 5.58, 95%CI 2.04–15.40). The MR analysis indicated that the use of statins, as genetically proxied, resulted in an increased risk of falling problems (OR 1.21, 95% CI 1.1–1.33).

**Conclusion:**

Our study found an association between the use of statins and an increased risk of balance problems and falls in adults over 40 years old, and the MR study result suggested statin use increased risk of falls. The risk was higher in participants under 65 years old compared to those over 65 years old.

## Highlights

• The problem of falls is a major threat to the health of the elderly population; statins can cause myopathy, suggesting that statins may cause balance problems and increase the risk of falling.
• Our cross-sectional study and Mendelian randomization analysis showed that statin use was associated with an increased risk of balance problems and falls (especially in adults aged between 40 and 65 years), and that genetically predicted statin use increased the risk of falling.
• The study suggested that adults aged 40–65 years who regularly use statins may need intensive assessments for fall risk.

## Introduction

Falls pose a significant health risk to the elderly population, often resulting in fall-related injuries such as fractures (Cuevas-Trisan, 2019). Medication prescription is a crucial factor in falls, as several commonly prescribed medications have been reported to increase the risk of falls (Dautzenberg et al., 2021; Lee et al., 2021; Seppala et al., 2021). It has been suggested that fall-risk-increasing drugs (FRIDs) should be deprescribed (Lee et al., 2021). Loop diuretics have been associated with an increased risk of falls (de Vries et al., 2018), while benzodiazepines were ranked as the most likely to cause falls in a European consensus study (Seppala et al., 2021). However, a systematic review found no robust evidence to support deprescribing FRIDs to prevent falls (Lee et al., 2021). The effectiveness of deprescribing FRIDs to prevent falls has been questioned due to uncertainty about the causal effect of a specific medication on fall risk.

The association between statin use and falls remains uncertain. While several studies and reviews have reported statin-associated myopathy, a condition characterized by muscle pain, muscle wasting, and muscle-related symptoms, it is unclear whether this condition increases the risk of falls. A 2023 study found that statin use was associated with increased stride time variability (Osman et al., 2023). Which is a marker of gait instability and may increase the risk of falls. However, Osman et al. stated that further study was needed regarding the finding that statin use increased stride time variability (Osman et al., 2023). Although statin use has been associated with an increased risk of falls and fall-related fractures in people over 80 years of age (Lazris and Roth, 2019), it has also been reported to reduce the risk of falls caused by digitalis and digoxin (de Vries et al., 2018). Two studies with traditional observational designs and relatively larger sample sizes than the others suggest that statin use may increase the risk of falls in elderly populations (Scott et al., 2009; Wang et al., 2020). However, due to the limited sample sizes and traditional observational design of these studies, a cause-and-effect relationship cannot be confirmed.

Mendelian randomization (MR) study is a quasi-experimental design that uses genetic instruments to infer causal relationship (Davies et al., 2018; Bowden and Holmes, 2019). The genetic instrument typically consists of SNPs, which are randomly assigned to individuals at conception. As a result, the population is naturally divided into two groups: those with SNP variation and those without. The MR design is less susceptible to confounding issues and is theoretically superior in making causal inferences. Therefore, it is increasingly utilized to identify causal relationships between phenotypes. Using the aforementioned background information, we conducted a cross-sectional study and an MR study to investigate the potential association between statin use and the risk of falls.

## Materials and methods

### Study design and setting

**FIGURE 1:**
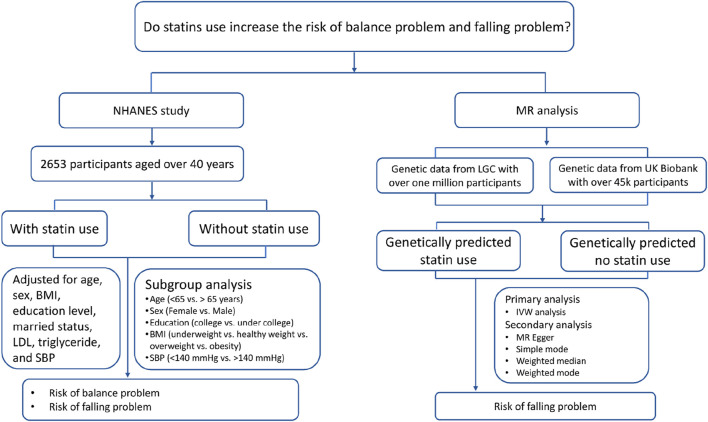
Study Design and Process Abbreviations: BMI, body mass index. LDL, low density lipoprotein. LGC, Lipid Genetics Consortium. MR, Mendelian randomization. NHANES, National Health and Nutrition Examination Survey. UK, United Kingdom. SBP, systolic blood pressure.

The research included a cross-sectional study incorporated within the National Health and Nutrition Examination Survey (NHANES) and a two-sample MR study. The National Center for Health Statistics Ethics Review Board approved the cross-sectional survey. The study utilized data from both the Lipids Genetic Consortium and the UK Biobank. Ethical approvals were secured from all participating centers in the primary analysis, and summary-level data was utilized, negating the need for any additional ethical approvals. The purpose of this cross-sectional study was to explore whether regular statin users had a greater likelihood of experiencing issues with balance and falls. The study aimed to investigate if genetically predicted statin use resulted in a higher incidence of falls. Figure 1 depicts the study design.

### Data collected from the NHANES

We obtained information on demographics, medical conditions, physical examination, and laboratory tests from the National Center for Health Statistics. Participants were eligible if they were at least 40 years of age, had reported balance or falling problems, and information of statin use. The data were collected by household interview. A trained household interviewer administrated these questions to the survey participant during the household interview. Participants were asked about the following problems: Have you ever had problems with balance problems/falling? Responses were categorized as yes, no, refused, do not know. Participants who answered yes or no were included in the final analysis.

Participants in the statin use category were identified from NHANES records collected during the household interview. During the interview, participants were asked if they had taken any prescription medications in the past month; those who answered “yes” were asked to show the interviewer all containers of the medications used. Prescription names were automatically matched to the Master Drug Database (MDDB^®^), a proprietary database of Facts and Comparison (Indianapolis, Indiana), to aid in data collection.

The 1999–2004 NHANES survey provided data on age, sex, BMI, blood pressure, education, marital status, muscle strength, and lipid levels, extracted from three rounds of the survey.

### Data sources for the MR study

We conducted a drug-target MR study, using the two-sample MR design. In our study, we used the five SNPs (rs12916, rs12173076, rs10515198, rs3857388, rs7711235) that have been shown to predict the effect of statins (Zhao et al., 2023). We acquired summary-level GWAS data from the Global Lipids Genetics Consortium and used low-density lipoprotein (LDL) levels as the biomarker to scale the effect of genetically predicted statin use (Graham et al., 2021). The Global Lipids Genetics Consortium recruited participants from five genetic ancestry groups: Admixed African or African (AdmAFR, N = 99.4 k, 6.0% of the sample), East Asian (EAS, N = 146.5 k, 8.9%), European (EUR, N = 1.32 m, 79.8%), Hispanic (HIS, N = 48.1 k, 2.9%), and South Asian (SAS, N = 41.0 k, 2.5%). The Global Lipids Genetics Consortium conducted a meta-analysis of genetic data from various study populations (Graham et al., 2021), and the resulting summary-level genetic data were made publicly available (http://csg.sph.umich.edu/willer/public/glgc-lipids2021/). Participants from the UK Biobank were excluded in the Global Lipids Genetics Consortium dataset to avoid sample overlap. For this study, genetic data from around 1.3 million participants were utilized for analysis. Prior research indicates that the five SNPs resulted in an LDL-lowering effect of 6.7 mmol/L decrease per SD (Zhao et al., 2023).

Genetic information regarding the risk of falling was acquired from a UK Biobank cohort that consisted of 89,076 cases reporting at least one fall within 1 year of the interview and 362,103 controls (Trajanoska et al., 2020). The number of falls reported in the UK Biobank was obtained using a touch-screen questionnaire. Participants were asked if they had experienced any falls in the past year. Those who answered affirmatively were classified as fall cases.

### Statistical analysis

Descriptive statistics were used to tabulate data in participants with and without a balance or fall problem. Categorical data was analyzed using frequencies and percentages, while continuous data was assessed using means and standard deviations (SDs).

To assess the potential link between statin use and increased risk of balance problems and falls, we conducted both univariate and multivariate logistic regression analyses. In these analyses, we designated balance problems and falls as dependent variables and statin use as the independent variable. To account for unequal probabilities of selection, oversampling, and non-response, we applied weights generated in the baseline variables (WTINT2YR - Full Sample 2 Year Interview Weight). In the multivariate logistic regression model, we also controlled for important covariates such as age, gender, body mass index, education level, marital status, LDL and triglyceride levels, and systolic blood pressure (SBP) levels. We conducted additional analyses on the variables that displayed statistical significance in the multivariate model, specifically age, sex, blood pressure, education level, and BMI.

A recent systematic review of risk factors associated with falls found that older age, lower education, and hypertension were linked to a higher risk of falls (Xu et al., 2022). Additionally, a meta-analysis revealed that postmenopausal women with a higher BMI had an increased risk of falls. Based on the two meta-analyses and our own research, we conducted subgroup analyses (Zhao et al., 2020). In the subgroup analyses, age was categorized as 40–65 years *versus* over 65 years. SBP level was categorized as less than 140 mmHg *versus* 140 mmHg or greater. Education level was classified as college or higher *versus* less than college. BMI was classified as underweight (BMI <18 kg/m^2^), healthy weight (BMI 18–24.9 kg/m^2^), overweight (BMI 25–29.9 kg/m^2^), and obesity (BMI >30 kg/m^2^). Forest plots were presented to show the associations between statin use and balance and fall problems in different subgroups.

The primary analysis for MR was conducted utilizing the IVW approach to combine the effects of five SNPs (mimicking statin use) on the probability of encountering falling difficulties. The consistency of the primary findings was verified by conducting secondary analyses, namely, weighted median, simple mode, weighted mode, and MR Egger.

The study determined the effect size of statin medication on balance and falling issues through calculation of odd ratios (OR), accompanied by the appropriate 95% confidence interval (95%CI) reporting. The analyses were conducted using R statistical software (version 4.2.2, www.r-project.org), and the TwosampleMR package (version 0.5.6) was utilized for the MR analysis.

## Results

### The cross-sectional study

#### Study population

The cross-sectional study included a total of 2,653 participants. The participants’ mean age was 65.5(SD, 13.9) years, with a mean BMI of 28.8 (6.6) kg/m^2^, a mean SBP of 135 (23.7) mmHg, and a mean time to complete a 20-foot walk of 8.3 (4.2) seconds. The mean LDL was 3.16 (1.02) mmol/L, and the mean triglyceride was 1.87 (1.35) mmol/L. Of the participants, 60% were female, 33.6% had attended college, and 18.4% were concomitant statin users. Participants who experienced issues with balance had a greater likelihood of using statins compared to those who did not face balance problems (19.6% *versus* 16.4%). Similarly, participants who experienced falls also had a higher rate of statin use than those who did not experience any falls (21.1% *versus* 17.4%). Further information on the study population is provided in Table 1.

**TABLE 1:** Baseline characteristics of participants from NHANES.

Items	With balance problem (n = 1728)	Without balance problem (n = 923)	All (n = 2,651)	With falling problem (n = 748)	Without falling problem (n = 1905)	All (n = 2,653)
Age at recruiting, yr	67.2 (13.7)	62.2 (13.6)	65.4 (13.9)	69.6 (13.3)	63.8 (13.8)	65.5 (13.9)
Female, n (%)	1,010 (58.4)	581 (62.9)	1,591 (60)	456 (61)	1,135 (59.6)	1,591 (60)
BMI, kg/cm^2^	28.8 (6.7)	28.9 (6.4)	28.8 (6.6)	29 (6.9)	28.7 (6.4)	28.8 (6.6)
SBP (mmHg)	135 (23.6)	135 (23.9)	135 (23.7)	136 (24.1)	134 (23.5)	135 (23.7)
DBP (mmHg)	68.6 (16.4)	71.4 (15.2)	69.6 (16)	67.6 (16.7)	70.3 (15.7)	69.6 (16)
Education level, n (%)						
Colleage level	583 (33.7)	307 (33.3)	890 (33.6)	223 (29.8)	669 (35.1)	892 (33.6)
High school level	409 (23.7)	217 (23.5)	626 (23.6)	188 (25.1)	438 (23)	626 (23.6)
Less than high school	719 (41.6)	399 (43.2)	1,118 (42.2)	325 (43.4)	793 (41.6)	1,118 (42.1)
Unclear	17 (1)	0 (0)	17 (64.1)	12 (1.6)	5 (0.26)	17 (0.6)
Marital status, n (%)						
Married	807 (46.7)	501 (54.3)	1,308 (49.3)	318 (42.5)	993 (52.1)	1,311 (49.4)
Widowed	451 (26.1)	175 (19)	626 (23.6)	229 (30.6)	397 (20.8)	626 (23.6)
Divorced	222 (12.8)	99 (10.7)	321 (12.1)	103 (13.8)	218 (11.4)	321 (12.1)
Separated	58 (3.4)	27 (2.9)	85 (3.2)	27 (3.6)	57 (3)	84 (3.2)
Never married	95 (5.5)	56 (6.1)	151 (5.7)	37 (4.9)	114 (6)	151 (5.7)
Live with partner	39 (2.3)	37 (4)	76 (2.9)	11 (1.5)	65 (3.4)	76 (2.9)
Other	56 (3.2)	28 (3)	84 (3.2)	23 (3.1)	61 (3.2)	84 (3.2)
Time to complete 20 feet walk, seconds	8.8 (4.6)	7.3 (3)	8.3 (4.2)	10.1 (5.3)	7.7 (3.4)	8.3 (4.2)
LDL (mmol/L)	3.18 (1.11)	3.13 (0.83)	3.16 (1.02)	3.16 (1.27)	3.16 (0.92)	3.16 (1.02)
Triglyceride (mmol/L)	1.89 (1.43)	1.82 (1.2)	1.86 (1.35)	1.99 (1.44)	1.83 (1.32)	1.87 (1.35)
Accompanied with statin use, n (%)	338 (19.6)	151 (16.4)	489 (18.4)	158 (21.1)	331 (17.4)	489 (18.4)

#### Univariate and multivariate logistic analysis

Table 2 presents findings from the univariate and multivariate logistic regression analyses. Statin utilization was significantly associated with an elevated risk of balance issues among both univariate (OR, 1.25; 95%CI, 1.02–1.55) and multivariate analyses (OR, 2.02; 95%CI, 1.13–3.73). Additionally, univariate analysis revealed an association between statin use and an increased risk of falls (OR, 1.27; 95%CI, 1.03–1.57). However, the multivariate analysis revealed a comparable odds ratio but an insignificant discovery due to a more extensive 95%CI (OR, 1.49; 95%CI, 0.79–2.72).

**TABLE 2:** The association between statin use and risk of falls.

	No. Reported (cases/controls)	Or (95%CI)	*p*-value
Univariate analysis			
Balance problem	2,651 (1728/923)	1.25 (1.02–1.55)	0.037
Falling problem	2,653 (748/1905)	1.27 (1.03–1.57)	0.026
Multivariate analysis			
Balance problem	2,651 (1728/923)	2.02 (1.13–3.73)	0.021
Falling problem	2,653 (748/1907)	1.49 (0.79–2.72)	0.206
MR analysis^a^			
Falling problem	451,179 (89,076/362,103)	1.21 (1.1–1.33)	<0.001

^a^ We performed a two-sample MR, analysis. The exposure data were obtained from the Lipids Genetics Consortium, in which we excluded the UKB, population, and we included the study population of European ancestry. The outcome data (fall risk) were obtained from the UKB, population. The inverse-variance weighted (IVW) method was used as the primary analysis to pool the outcomes, and the result of the IVW, analysis is presented in Table 2, while the results of the secondary analyses are presented in Supplementary Figure S1 in the Supplement.

#### Subgroup analysis

**FIGURE 2:**
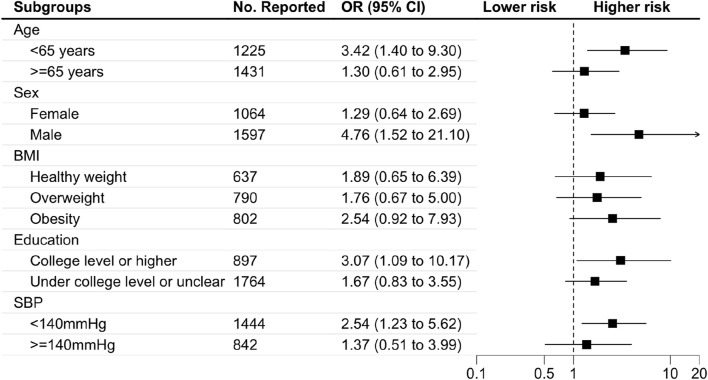
Subgroup analysis of the association between statin use and balance problem Abbreviations: BMI, body mass index. OR, odds ratio. SBP, systolic blood pressure. Footnote: Subgroup analysis showed that participants who were younger than 65 years, male, college or higher education, and SBP <140 mmHg had a higher risk of balance problems.

**FIGURE 3:**
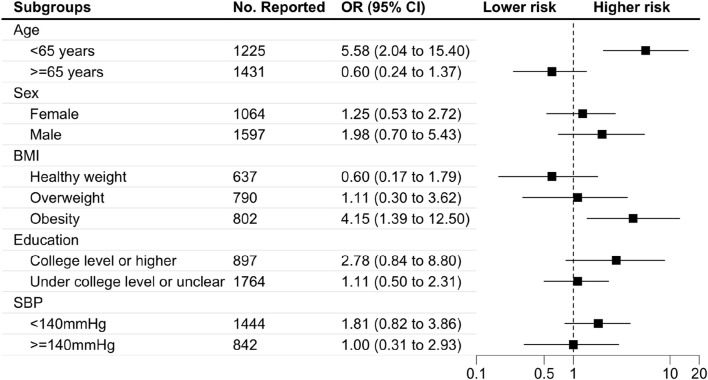
Subgroup analysis of the association between statin use and falling problem Abbreviations: BMI, body mass index. OR, odds ratio. SBP, systolic blood pressure. Footnote: The subgroup analysis showed that participants who were younger than 65 years and obese had a higher risk of falling problems.

Figures 2, 3 display the findings of the subgroup analyses. The risk of balance problems was greater in participants aged 40–65 years, male participants, those with a college education or higher, and those with SBP <140 mmHg than in participants aged ≥65 years, female participants, those with a college education or lower, and those with SBP >140 mmHg. Participants aged 40–65 years with obesity have a higher risk of falling than those over 65 years and those with a healthy weight or overweight. We performed analyses to detect the association between statin use and subgroups, and the results showed that statin use was significantly associated with older age, higher proportion of males, and higher BMI index.

#### The MR analysis

Table 2 presents the findings of the MR analysis. According to the IVW analysis, statin utilization increased the risk of a fall problem (OR 1.21, 95%CI, 1.1–1.33). Supplementary Figure S1 (in the Supplement) shows the results of the sensitivity analyses, which yielded results comparable to those of the IVW analysis. Supplementary Figure S2 (in the Supplement) shows the influence of each SNP that inhibits 3-hydroxy-3-methylglutaryl-CoA reductase (HMGCR) function (simulating statin use) on the risk of falling, and shows that the largest effect size belongs to SNP rs12916 (OR, 1.23; 95%CI, 1.09–1.4).

## Discussion

It is unclear whether statin use increases the risk of falling problems in the adult population. Our study adds to the evidence that statin use is associated with an increased risk of balance problems and falling problems based on NHANES survey data. Further subgroup analysis showed that younger age, lower SBP, college education, and being female were associated with a higher risk of balance problems, and younger age and obesity were associated with a higher risk of falling problems. Further MR analysis showed that statin use increased the risk of falling.

Statin use has been reported to induce myopathy (Lazris and Roth, 2019; Ward et al., 2019; Dicken et al., 2022), so it may increase the risk of falling problems. No randomized controlled trials have been conducted to examine whether statin use increases the risk of falling problems. This may be partly due to the low rate of myopathy reported in RCTs evaluating the efficacy of statins, and it may also be due to under-reporting of myopathy or muscle symptoms in RCTs, as observational studies reported a higher rate of myopathy caused by statin use (10%–29% in observational studies *versus* 9.4% in RCTs). However, myopathy may not be the sole reason for statins causing an increased risk of falls. A study utilizing MR methods failed to detect a causal effect of statin use on myopathy (Liu et al., 2021), which contradicts our findings. The discrepancies between their study and ours are that they used data from sources other than the UK Biobank and used myopathy as an outcome instead of fall risk, which explains the inconsistent findings between the two studies. We used a cross-sectional design and an MR design to try to answer this question. The cross-sectional study was used to detect the association between statin use and the risk of balance problems and falls, and the MR analysis was used to determine whether the association was causal, because the MR design was considered a quasi-experimental design. The results of the two designs were consistent and showed that statin use may increase the risk of falls.

Although our study suggests that statin use increases the risk of balance problems and falls, it is still unrealistic to translate this information into clinical practice. Statin use is important in the prevention of cardiovascular and cerebrovascular events, so it is unrealistic and unethical to discontinue statin use. Future studies are warranted to determine which type of statin and dose is associated with the highest risk of falls. The FDA report on rhabdomyolysis from 1990 to 2002 showed that fluvastatin had the lowest rate of rhabdomyolysis (1.6%) and that cerivastatin had the highest rate of rhabdomyolysis (56.9%) (Thompson et al., 2003). The report showed a wide variation in the rates of rhabdomyolysis - an important myopathy - caused by statins, suggesting that the rates of adverse effects vary between statins. Clarifying which statin caused the highest rate of myopathy that could lead to a falling problem will help develop a medication strategy to reduce the risk of a falling problem.

Statin-associated myopathy may partly explain the increased risk of falling; another explanation for our study result should also be considered: statins are commonly used in patients with hyperlipidemia, which is also strongly associated with balance problems (Sfakianaki et al., 2021). Hyperlipidemia causes lesions in the cerebral vessels and increases the risk of cerebral hypoperfusion. Hypoperfusion in the brainstem and cerebellum increases the rate of dizziness (Li et al., 2016), and dizziness is a common type of balance problem that increases the risk of falls (Sfakianaki et al., 2021). In addition, participants with hyperlipidemia had a recurrence rate of benign paroxysmal positional vertigo (BPPV) of 67.8% (Sfakianaki et al., 2021), and BPPV is a known condition characterized by dizziness and closely associated with an increased risk of falls (Liao et al., 2015). However, our MR analysis did not support this explanation because MR analysis uses genetic tools to infer causality and is less susceptible to confounding than traditional observational studies (Bowden and Holmes, 2019).

Our study also showed an interesting finding in the subgroup analysis. Statin-associated falls were more frequent in participants younger than 65 years than in those older than 65 years. This finding may suggest that deprescribing statins or limiting statin use is not necessary in the elderly population over 65 years of age, because in this population, the risk of falling and balance problems showed no statistical difference between participants using statins and those not using statins. However, the mechanism behind this finding was unclear. Statin-associated myopathy was unlikely to be the cause because myopathy usually occurs in older participants. Regarding the subgroup analyses performed only in the survey study, we could not rule out the possibility that participants who used statins between the ages of 40 and 65 years were affected by hyperlipidemia in their early life, because MR analysis was not performed in this subgroup and the causal relationship could not be confirmed. Younger patients with hyperlipidemia may be associated with longer exposure to vascular lesions correlated with hyperlipidemia, and a high rate of vertigo due to cerebrovascular hypoperfusion would be expected in this population.

Our study had limitations. Firstly, it may have encountered internal validity issues. We assessed the causal effect of statin use on fall risk in the MR study, but there is still a possibility of reverse causality. We conducted a two-sample MR study with summary-level data, which could not control for important confounding issues due to the lack of individual participant data. Secondly, there may be construct validity issues. Both the cross-sectional survey and MR study relied on self-reported falls outcomes, which may not accurately reflect the severity of the falls. The most important outcome to avoid in the elderly population is falls resulting in fractures. In 2018, fall-related injuries among older adults in the US population resulted in approximately 32,000 deaths (Moreland et al., 2020). This finding indicates that future studies should include the classification of the severity of falls. It is also important and urgent to identify whether statin use causes a higher rate of falls related fractures. Third, the study’s external validity should be further examined. The analysis did not include an evaluation of which type of statins was associated with the highest rate of falling problems, making it difficult to develop a strategy for preventing falling problems based on our study results. Additionally, the NHANES survey did not assess the frequency and duration of statin use, preventing us from evaluating the effect of statin use duration on the study results. Data from a predominantly European ancestry population was used, which may limit the generalizability of the study results. It is unclear whether statin use is associated with higher rates of balance problems and falling in other populations, such as Asian populations, and further studies are needed. Fourth, In conclusion, our study demonstrated an association between statin use and an increased risk of balance problems and falls, and MR analysis confirmed that statin use had a causal effect on the risk of falls. However, the clinical significance of the findings in the development of fall prevention strategies is still unclear because the mechanisms of statin-associated falls were not specified and whether the type, duration, and frequency of treatment had an effect on the risk of falling problems.

## Data Availability

The datasets presented in this study can be found in online repositories. The names of the repository/repositories and accession number(s) can be found below: https://wwwn.cdc.gov/nchs/nhanes/.
