# Supplementary material for: Statin use and fall risk in adults: a cross-sectional survey and mendelian randomization analysis
Source: Front Pharmacol. 2024 Jun 26;15:1364733. doi: 10.3389/fphar.2024.1364733 (PMC11233697; doi:10.3389/fphar.2024.1364733)
Supplement: Supplementary file 1 [file DataSheet1.DOCX]

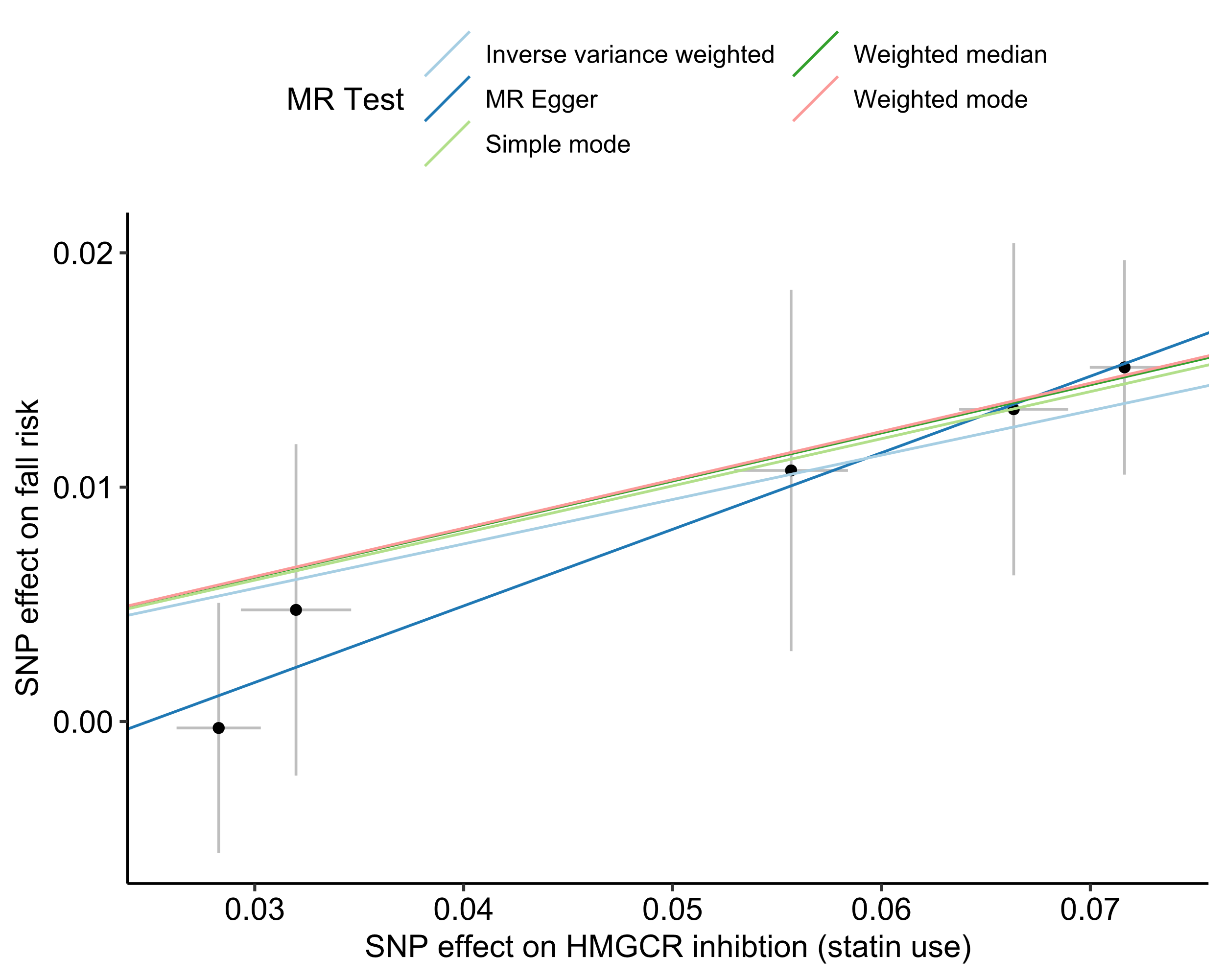


**Figure S1. Sensitivity analyses of the Mendelian randomization study**

*Abbreviations*: MR, Mendelian randomization. SNP, single nucleotide polymorphisms. HMGCR, 3-hydroxy-3-methylglutaryl-CoA reductase.

*Footnote:* The primary analysis was inverse variance weighted analysis, and weighted median, MR Egger, Weighted mode, and simple mode were treated as sensitivity analyses.


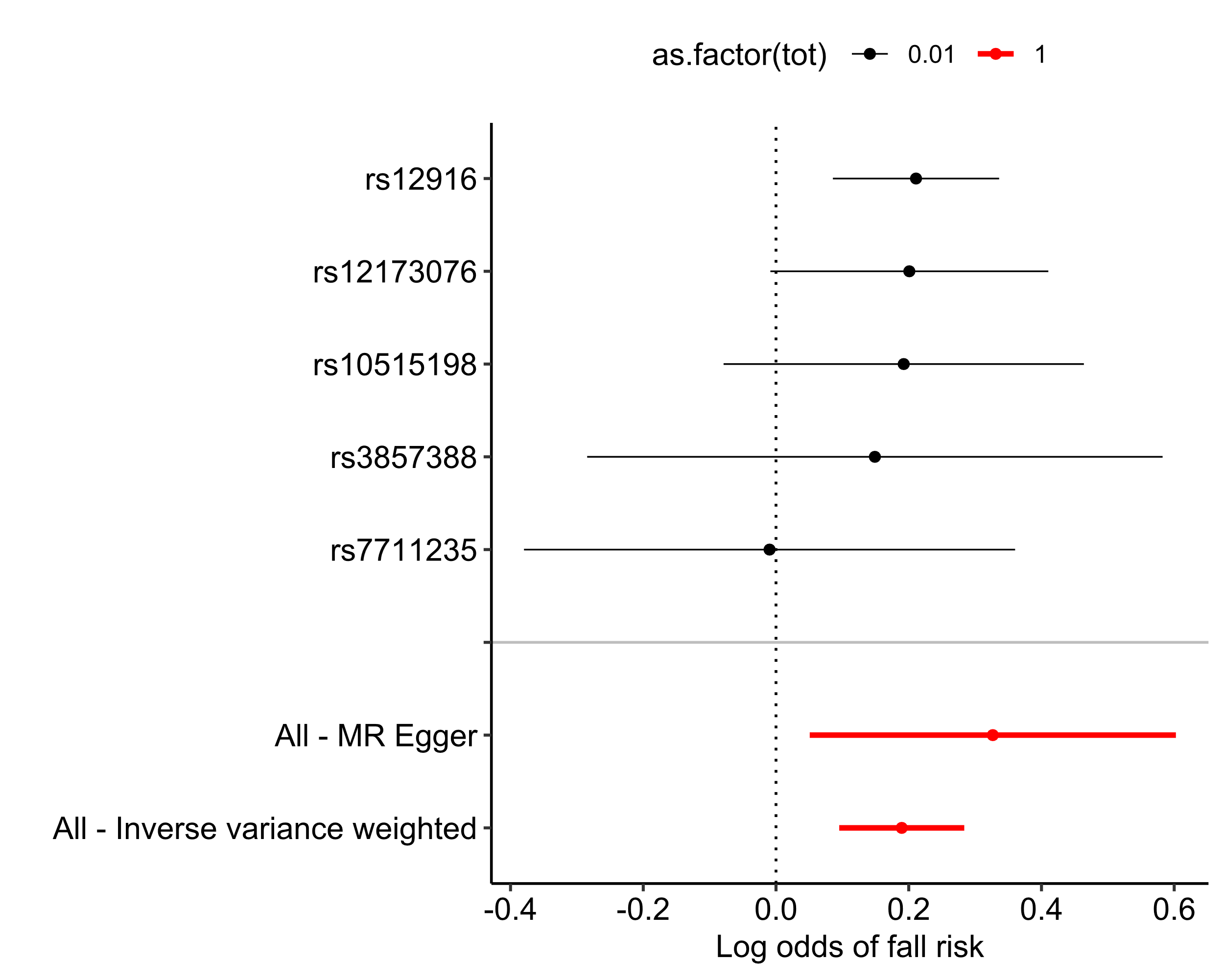


**Figure S2. The effect of each SNP predicting statin use on fall risk**

*Abbreviations*: MR, Mendelian randomization. SNP, single nucleotide polymorphisms. HMGCR, 3-hydroxy-3-methylglutaryl-CoA reductase.

*Footnote:* The Y-axis lists the names of the SNPs. The SNP, rs12916, had the largest effect size.
